# Supplementary material for: TECTB Variants Reveal Tectorial Membrane Vulnerability in Dominant Non‐Syndromic Hearing Loss
Source: Adv Sci (Weinh). 2026 Sep 6:e77543. Online ahead of print. doi: 10.1002/advs.77543 (PMC13547704; doi:10.1002/advs.77543)
Supplement: Supplementary file 1 [file ADVS-9999-e77543-s001.pdf]

TECTB Variants Reveal Tectorial Membrane Vulnerability in Dominant Non-Syndromic Hearing Loss

Evan B. Hale[1,2,3], Barbara Vona[4,5,6,#], Richard J. Goodyear[7], Richard T. Osgood[1,2], Jun Shen[8,9], Sami S. Amr[8,9,10], Karen Mojica[11], Michaela A. H. Hofrichter[12], Jörg Schröder[13], Sophie Flandin[14], Paul Gratias[1,2], Ricardo Vera-Monroy[15], Katherine Callahan[15], Kerry L. Gudlewski[15], Rolen Quadros[16], Masato Ohtsuka[17], JoAnn McGee[18], Edward J. Walsh[18], Cynthia C. Morton[8,9], Thomas Haaf[13], Channabasavaiah Gurumurthy[16], James E. Saunders[15], Guy P. Richardson[7,#], Artur A. Indzhykulian[1,2,3,#].

1. Mass Eye and Ear, Eaton Peabody Laboratories, Boston, MA, 02114, USA.
2. Department of Otolaryngology Head and Neck Surgery, Harvard Medical School, Boston, MA, 02115, USA.
3. Speech and Hearing Bioscience and Technology Program, Harvard University, Cambridge, MA, 02138, USA.
4. Institute for Auditory Neuroscience and InnerEarLab, University Medical Center Göttingen, 37075 Göttingen, Germany.
5. Auditory Neuroscience and Optogenetics Laboratory, German Primate Center, 37077 Göttingen, Germany.
6. Collaborative Research Center 1690 (CRC1690), University of Göttingen, 37075 Göttingen, Germany.
7. Sussex Neuroscience, School of Life Sciences, University of Sussex, Falmer, Brighton, UK.
8. Department of Pathology, Brigham and Women's Hospital, Boston, MA, 02115, USA.
9. Department of Pathology, Harvard Medical School, Boston, MA, 02115, USA.
10. Laboratory for Molecular Medicine, Mass General Brigham Personalized Medicine, Cambridge, MA, 02115, USA.
11. Department of Otolaryngology, Vivian Pellas Hospital, Managua, Nicaragua.
12. Institute of Clinical Genetics and Genome Medicine, University Hospital Würzburg, Würzburg, Germany.
13. Institute of Human Genetics, Julius-Maximilians-University Würzburg, Würzburg, Germany.
14. Department of Otolaryngology, Comprehensive Hearing Center, University Clinics, Würzburg, Germany.
15. Section of Otolaryngology-Head and Neck Surgery, Department of Surgery, Dartmouth-Hitchcock Medical Center, Geisel School of Medicine at Dartmouth, NH, 03766, USA.
16. Mouse Genome Engineering Core Facility, Nebraska Medical Center, Omaha, NE, 68198, USA.
17. Division of Basic Medical Science and Molecular Medicine, School of Medicine, Tokai University, Kanagawa, Japan
18. Developmental Auditory Physiology Laboratory, Boys Town National Research Hospital, Omaha, NE, 68131, USA

[#]Correspondence should be addressed to:

Dr. Artur A. Indzhykulian Artur_Indzhykulian@meei.harvard.edu
Prof. Guy P. Richardson G.P.Richardson@sussex.ac.uk
Dr. Barbara Vona Barbara.Vona@med.uni-goettingen.de

Supplementary Tables

Supplementary Table 1. Summary of known tectorial membrane-expressed genes

Gene	DFN locus	Inheritance	Human phenotypes	References
CEACAM16	DFNA4B<br>DFNB113	AD<br>AR	Progressive mild-to-moderately-severe SNHL<br>Progressive mild-to-moderate SNHL	PMID: 21368133<br>PMID: 29703829
OTOA	DFNB22	AR	Moderate to severe mid-frequency SNHL	PMID: 11972037
OTOG	DFNB18B	AR	Mild-to-moderate SNHL	PMID: 23122587
OTOGL	DFNB84B	AR	Mild-to-moderate SNHL	PMID: 23122586
TECTA	DFNA8/12<br>DFNB21	AD<br>AR	Moderate to severe, stable or progressive mid-to-high-frequency SNHL; U-shaped audiograms<br>Moderate-to-severe SNHL; flat or U-shaped audiograms	PMID: 9590290<br>PMID: 9949200
TECTB	Current study	AD	Variable mild-to-severe SNHL	Current study

Abbreviations: Autosomal dominant, AD; autosomal recessive, AR; SNHL, sensorineural hearing loss

Supplementary Table 2. Risk Factor Questionnaire

Pedigree ID	Affected Status	Prior Audio	Maternal Infection	Med/Alcohol Exposure	Gentamycin Exposure	Pesticides	Maternal Tobacco	Prematurity	Apnea	Oxygen	Birth Defects	Visual Problems	Hematuria	Neck Mass	Otitis Media	Syncope	Balance Problems	Otorrhea	Treated for Otitis Media	Noise Exposure (TTS)	Occupational Noise
II:1	Unaffected	0	0	0	0	0	0	0	0	0	0	1	0	0	0	0	0	0	0	Never	0
II:2	Unaffected	0	0	0	0	0	0	0	0	0	0	0	0	0	0	0	0	0	0	Never	0
II:3	Unaffected	1	0	0	0	1	0	0	0	0	0	1	0	0	0	0	0	0	0	Never	0
II:4	Affected	1	NA	0	NA	0	1	0	NA	NA	0	0	0	0	0	0	1	0	0	Sometimes	0
II:5	Affected	1	NA	0	0	1	0	0	0	0	0	0	0	1	1	0	1	0	1	Never	0
III:1	Unaffected	1	0	0	0	1	0	1	0	0	0	0	0	0	0	0	0	0	0	Never	1
III:2	Affected	1	0	0	0	1	0	0	0	0	0	0	0	0	0	0	0	0	0	Never	0
III:3	Affected	1	0	0	0	0	0	0	0	0	0	0	0	0	0	1	1	0	0	Never	0
III:5	Affected	0	0	0	0	1	0	0	0	0	0	0	0	0	0	0	0	0	0	Never	0
III:6	Affected	1	0	0	0	1	0	0	0	0	0	0	0	0	0	0	1	0	0	Never	0
III:7	Unaffected	0	0	0	0	0	0	0	0	0	1	1	0	0	0	0	0	0	0	Sometimes	0
IV:1	Affected	0	0	0	0	0	0	0	0	0	0	0	0	0	0	0	0	0	0	*Shorter survey	
	Total N	12.0	10.0	12.0	11.0	12.0	12.0	12.0	11.0	11.0	12.0	12.0	12.0	12.0	12.0	12.0	12.0	12.0	12.0	11.0	11.0
	Positive	7.0	0.0	0.0	0.0	6.0	1.0	1.0	0.0	0.0	1.0	3.0	0.0	1.0	1.0	1.0	4.0	0.0	1.0	2.0	1.0
	Percentage	58.3	0.0	0.0	0.0	50.0	8.3	8.3	0.0	0.0	8.3	25.0	0.0	8.3	8.3	8.3	33.3	0.0	8.3	18.2	9.1

Abbreviations: NA, not answered; TTS, temporary threshold shift

Supplementary Table 3. Other variants identified in the exome sequencing data in the proband.

						Max. MAF gnomAD (v4.1)		All-of-Us							ACMG Criteria/ Classification	In linked interval?	
Gene	Transcript	cDNA (c.)	Protein Change (p.)	Zyg	Read Count (Variant:WT)	AF	AF Population	MAF	SIFT	PolyPhen-2	FATHMM	Mutation Taster	REVEL	CADD			Exclusion criteria
BCS1L	NM_001371449.1	c.670C>T	p.(Arg224Cys)	Het	520 (226:294)	3.1e-5	European (non-Finnish)	8.0e-6	0.00[1]	1.0[1]	-2.61[1]	0.99[1]	0.778[1]	32[1]	PM2_P, PP3_M/ VUS	No	Not in linked interval; Gene is associated with recessive disorders
DIS3L2	NM_152383.5	c.2074A>C	p.(Asn692His)	Het	119 (63:56)	2.2e-5	European (non-Finnish)	Not present	0.03[1]	0.994[1]	0.87[3]	0.33[3]	0.433[2]	28.6[1]	PM2_P/VUS	No	Not in linked interval; Gene is associated with a recessive disorder
GLYR1	NM_032569.4	c.1141C>T	p.(Arg381Cys)	Het	67 (29:38)	3.4e-6	European (non-Finnish)	4.0e-6	0.00[1]	1.0[1]	-0.24[2]	0.80[1]	0.67[1]	32.0[1]	PM2_P, PP3_P/ VUS	No	Not in linked interval; IMPC mouse with normal hearing
ITIH2	NM_002216.3	c.1223T>C	p.(Leu408Ser)	Het	58 (29:29)	2.2e-5	East Asian	4.0e-6	0.03[1]	1.0[1]	-1.49[2]	0.39[3]	0.71[1]	25.4[1]	PM2_P, PP3_P/ VUS	No	Not in linked interval
PLCB2	NM_004573.3	c.431G>T	p.(Arg144Leu)	Het	201 (115:86)	1.7e-4	Admixed American	2.0e-6	0.02[1]	0.954[1]	0.93[3]	0.59[3]	0.30[2]	26.8[1]	PM2_P/VUS	No	Not in linked interval
SLC10A2	NM_000452.3	c.724_725insC	p.(Phe242Serfs*5)	Het	44 (19:25)	0	--	0	--	--	--	--	--	--	PM2_P/VUS	No	Not in linked interval; Gene is associated with a recessive disorder
SMARCA4	NM_001387283.1	c.3355C>T	p.(Arg1119Cys)	Hom	725 (725:0)	8.5e-7	European (non-Finnish)	0	0.00[1]	1.0[1]	-1.07[2]	0.99[1]	0.89[1]	31[1]	PM2_P, PP3_M/ VUS	No	Not in linked interval; Gene is associated with a dominant disorder, phenotype mismatch
STON1	NM_006873.4	c.2167G>A	p.(Gly723Arg)	Het	51 (18:33)	0	--	-0	0.01[1]	1.0[1]	2.94[3]	0.19[3]	0.30[2]	31[1]	PM2_P/VUS	No	Not in linked interval;
*SUPV3L1**	NM_003171.5	c.1925G>A	p.(Ser642Asn)	Het	48 (29:19)	5.0e-5	Admixed American	2.2e-5	0.0[1]	1.0[1]	1.03[3]	0.93[1]	0.47[2]	35[1]	PM2_P	No	Not in linked interval;
TRPV2	NM_016113.5	c.1837C>T	p.(Gln613*)	Het	192 (106:86)	1.7e-5	Admixed American	1.2e-5	--	--	--	--	--	37[1]	PM2_P	No	Not in linked interval; Gene associated with perception to pain caused by heat
UBXN2B	NM_001077619.2	c.274G>A	p.(Ala92Thr)	Het	43 (18:25)	1.7e-5	Admixed American	6.0e-6	0.00[1]	1.0[1]	0.22[3]	0.54[3]	0.55[1]	27.6[1]	PM2_P/VUS	No	Not in linked interval; IMPC mouse with normal hearing
USP3	NM_006537.4	c.11C>G	p.(Pro4Arg)	Het	167 (94:73)	0	--	0	0.00[1]	0.45[3]	-1.81[1]	0.26[3]	0.63[1]	23.8[2]	PM2_P, PP3_P/ VUS	No	Not in linked interval; Unknown function

Pathogenicity is represented as [1]deleterious, [2]neutral, or [3]benign prediction, whereas “--” represents variant not scored.
Abbreviations: IMPC, International Mouse Phenotyping Consortium; VUS, variant of uncertain significance; Zyg, zygosity
*Predicted to cause aberrant splicing

Supplementary Table 4. List of genes included on the *OtoGenome* hearing loss gene panel

Gene	OMIM #	Gene	OMIM #	Gene	OMIM #	Gene	OMIM #
ACTG1	102560	*GJB2*	121011	*MTTS1*	590080	*SLC17A8*	607557
ADGRV1	602851	*GJB3*	603324	*MYH14*	608568	*SLC26A4*	605646
ATP6V1B1	192132	*GJB6*	604418	*MYH9*	160775	*SLC26A5*	604943
BSND	606412	*GPSM2*	609245	*MYO15A*	602666	*STRC*	606440
CCDC50	611051	*GRHL2*	608576	*MYO1A*	601478	*TECTA*	602574
CDH23	605516	*GRXCR1*	613283	*MYO3A*	606808	*TIMM8A*	300356
CLDN14	605608	*HGF*	142409	*MYO6*	600970	*TJP2*	607709
CLRN1	606397	*ILDR1*	609739	*MYO7A*	276903	*TMC1*	606706
COCH	603196	*KCNE1*	176261	*OTOA*	607038	*TMIE*	607237
COL11A2	120290	*KCNQ1*	607542	*OTOF*	603681	*TMPRSS3*	605511
CRYM	123740	*KCNQ4*	603537	*PCDH15*	604487	*TPRN*	613354
DFNA5	608798	*LHFPL5*	609427	*PDZD7*	612971	*TRIOBP*	609761
DFNB31	607084	*LOXHD1*	613072	*PJVK*	610219	*USH1C*	605242
DIAPH1	602121	*LRTOMT*	612414	*POU3F4*	300039	*USH1G*	607696
ESPN	606351	*MARVELD2*	610572	*POU4F3*	602460	*USH2A*	608400
ESRRB	602167	*MIR183*	611608	*PRPS1*	311850	*WFS1*	606201
EYA1	601653	*MIR96*	611606	*PTPRQ*	603317		
EYA4	603550	*MSRB3*	613719	*RDX*	179410		
GIPC3	608792	*MTRNR1*	561000	*SERPINB6*	173321		
